# Auto-titrating versus fixed continuous positive airway pressure for the treatment of obstructive sleep apnea: a systematic review with meta-analyses

**DOI:** 10.1186/2046-4053-1-20

**Published:** 2012-03-08

**Authors:** Stanley Ip, Carolyn D'Ambrosio, Kamal Patel, Ndidiamaka Obadan, Georgios D Kitsios, Mei Chung, Ethan M Balk

**Affiliations:** 1 Institute of Clinical Research and Health Policy Studies, Tufts University School of Medicine, Tufts Medical Center, Boston, MA, USA; 2 Center for Sleep Medicine, Tufts Medical Center, Boston, MA, USA

## Abstract

**Background:**

Obstructive sleep apnea is a relatively common disorder that can lead to lost productivity and cardiovascular disease. The form of positive airway treatment that should be offered is unclear.

**Methods:**

MEDLINE and the Cochrane Central Trials registry were searched for English language randomized controlled trials comparing auto-titrating positive airway pressure (APAP) with continuous positive airway pressure (CPAP) in adults with obstructive sleep apnea (inception through 9/2010). Six researchers extracted information on study design, potential bias, patient characteristics, interventions and outcomes. Data for each study were extracted by one reviewer and confirmed by another. Random effects model meta-analyses were performed for selected outcomes.

**Results:**

Twenty-four randomized controlled trials met the inclusion criteria. In individual studies, APAP and fixed CPAP resulted in similar changes from baseline in the apnea-hypopnea index, most other sleep study measures and quality of life. By meta-analysis, APAP improved compliance by 11 minutes per night (95% CI, 3 to 19 minutes) and reduced sleepiness as measured by the Epworth Sleepiness Scale by 0.5 points (95% CI, 0.8 to 0.2 point reduction) compared with fixed CPAP. Fixed CPAP improved minimum oxygen saturation by 1.3% more than APAP (95% CI, 0.4 to 2.2%). Studies had relatively short follow-up and generally excluded patients with significant comorbidities. No study reported on objective clinical outcomes.

**Conclusions:**

Statistically significant differences were found but clinical importance is unclear. Because the treatment effects are similar between APAP and CPAP, the therapy of choice may depend on other factors such as patient preference, specific reasons for non-compliance and cost.

## Background

Obstructive sleep apnea (OSA) is a relatively common disorder in the US. The Wisconsin Sleep Cohort Study, a prospective natural history study, reported that about 10% of adults aged 30 to 60 years old had clear evidence of OSA in 1988, when the study began [1]. A National Sleep Foundation poll in 2005 suggested that as many as one in four American adults are at high risk of OSA and could benefit from an evaluation for OSA [2].

The defining characteristic of OSA is a partial or complete obstruction of the airway while sleeping. The most common first-line therapy is the use of continuous positive airway pressure (CPAP) devices during sleep. The CPAP machine directly relieves the airway obstruction by increasing luminal pressure, thereby splinting the airway open. When used properly and consistently, CPAP results in improved sleep patterns and quality of life due to decreased daytime somnolence. However, many patients refuse the offer of CPAP therapy, do not tolerate it or fail to use CPAP devices properly [3]. Patients commonly did not fully comply with CPAP use, either using the device for only part of the night or only on some nights. This non-compliance has fueled the development of a number of technological solutions.

The most common variation on delivering positive airway pressure is the use of auto-titrating positive airway pressure (APAP) devices. Fixed CPAP provides continuous fixed pressure during the entire sleep period. In contrast, APAP varies the pressure delivered depending on changes in airflow resistance. Such changes in airflow resistance during sleep are dependent on many factors like posture and the degree of nasal congestion. Theoretically, varying the pressure delivered would promote an increase in breathing synchrony with the CPAP device and therefore could improve patient comfort with the device and thus enhance compliance. A 2009 Cochrane review comparing APAP with CPAP concluded that APAP was slightly more efficacious than CPAP in increasing patient compliance [4]. We undertook the present review in the context of a larger review of all primary studies of treatments for OSA. We sought to update and expand upon the previous review. The aim of the present review is to evaluate the body of evidence regarding the comparative efficacy of APAP versus CPAP on clinical and sleep-related outcomes, quality of life, compliance and other outcomes.

## Methods

We followed standard systematic review methods as described in the Agency for Healthcare Research and Quality (AHRQ) Methods Reference Guide for Effectiveness and Comparative Effectiveness Reviews [5]. A full technical report describing these methods in detail, including literature search strategies, and presenting our findings in full (with evidence tables) is available elsewhere [6].

We searched the MEDLINE and Cochrane Central Trials Registry databases from study inception to September 2010 for English language studies examining adults (older than 16 years) with OSA. Our search, available in the full technical report [6], included terms for OSA, sleep apnea treatments and relevant research designs. The full literature search was performed for a range of key questions about OSA diagnosis, treatment with any intervention and predictors of outcomes. Six reviewers independently screened the abstracts. We used a computerized screening program, abstrackr, to automate the screening of abstracts for the selection of eligible articles for full-text screening [7]. The abstrackr software uses an active learning algorithm to screen for relevant articles. Relevance was established by manually double-screening 1,000 abstracts to train the program. Subsequently, abstracts selected by the program were screened by one researcher. The results of screening were iteratively fed into the program for further training. This process continued until the program was left with only abstracts it rejected. Using abstrackr, we reduced by 50% the number of abstracts we needed to manually screen prior to starting the subsequent steps of the systematic review. Later, all abstracts rejected by abstrackr were manually screened for confirmation and were eventually rejected. Full-text articles were rescreened for eligibility by the same six reviewers.

We included peer reviewed, randomized controlled trials (RCTs) that compared APAP with fixed CPAP in ≥10 patients per intervention with confirmed diagnoses of OSA, including a formal sleep study demonstrating an apnea-hypopnea index (AHI) ≥5 events/hour. We included studies of any duration, though CPAP had to be used by the patients at home. Outcomes of interest included: objective clinical outcomes (death, cardiovascular events, hypertension, non-insulin dependent diabetes, depression); sleep and wakefulness related clinical outcomes (quality of life, sleepiness measures, neurocognitive tests, accidents, productivity); sleep study measures (AHI, arousal index, deep sleep, sleep efficiency, minimum oxygen saturation); comorbidity intermediate outcomes (hemoglobin A1c, blood pressure); compliance; and adverse events or harms.

Data from each study were extracted by one of six reviewers and confirmed by another. Extracted data included information on study and patient characteristics, details concerning the CPAP devices used, outcomes and study quality. For most outcomes, only data from the last reported time-point were included. We assessed the methodological quality of each study on the basis of predefined criteria in accordance with AHRQ's suggested methods for systematic reviews [5]. The primary data extractor determined the study quality (rated with the letter grades A, B or C), and at least one other reviewer confirmed it. Quality A studies adhered most closely to the commonly held precepts of high quality, including clear descriptions of the population, setting, interventions, outcomes and design; no obvious reporting omissions or errors; fewer than 20% dropouts; and no obvious source of bias. Quality B studies had some deficiencies in these criteria that were, however, unlikely to engender a major bias. Quality C studies had inadequate descriptions of their studies or had substantial flaws in reporting or design, such that a major bias could not be excluded.

We performed random effects model meta-analyses of differences of selected continuous variables between interventions where there were at least three unique similar studies [8]. Based on available data and our *a priori *assessment of the clinical importance of specific outcomes, we performed meta-analyses for the AHI, the Epworth Sleepiness Scale (ESS), arousal index (per hour frequency of arousals from sleep), minimum oxygen saturation (during sleep), the multiple sleep latency test (measurement of how quickly a subject will fall asleep during the day), the quality of life measure Functional Outcomes Sleep Questionnaire and compliance (measured as time per night using the device). When necessary, standard errors of the net change (difference between the within-arm changes) were calculated from CIs, *P *values or from the standard errors of the within-arm changes. When necessary, standard errors of the within-arm changes were estimated from the standard errors of the baseline and final values, assuming a 50% correlation between the two. Studies that compared two different forms of APAP to CPAP were treated as independent despite the common CPAP arm. Due to limitations of the reported data and for consistency, in cross-over studies we treated the difference in final values as equivalent to the net change, under the assumption that the baseline values were equal and would thus cancel out. Heterogeneity among effect sizes was assessed using the I^2 ^index, and the chi-square test. An I^2 ^index ≥50% was used to indicate medium-to-high heterogeneity [9].

To explore sources of heterogeneity in between-study findings, all forest plots were drawn with subgroup meta-analyses of trials stratified by baseline OSA severity (as determined by the minimum AHI threshold required in each study for the diagnosis of OSA). Forest plots sub-divided by study design are presented in the full technical report [6]. The decision to subgroup studies by minimum AHI and by study design was made *a priori*; however, the minimum AHI categories were based on thresholds reported in the studies. We performed meta-regressions separately with AHI thresholds and study design to determine statistically significant differences among subgroups.

We graded the strength of the body of evidence based on the AHRQ Methods Reference Guide [5]. We took into account the overall study quality, the consistency across studies, the applicability of the studies to the general population of patients treated for OSA, the magnitude and precision of the treatment effects and the relative clinical importance of the different outcomes assessed [6]. The overall strength of evidence was rated as high, moderate, or low - which each indicate the level of confidence that the evidence reflects the true effect - or insufficient.

## Results

**Figure 1:**
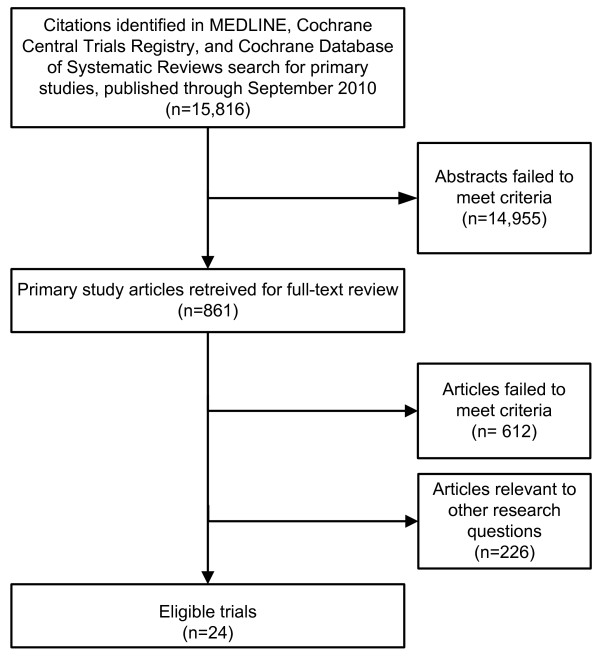
**Literature selection flow**.

Our literature search yielded 15,816 citations, from which 861 articles were retrieved (Figure 1). We identified 24 RCTs that compared APAP with fixed CPAP treatment in patients with OSA **(**Table 1; [10-33]). Three RCTs [31-33] identified in prior meta-analyses [4,34] were added after completion of our full technical report [6]. Fifteen trials used a cross-over design and nine a parallel design. Studies generally failed to report complete data about outcomes. For 17 studies, the variance of the difference in baseline and final values was not reported and had to be estimated by making an assumption about the correlation between the values. Patients who were new to positive airway pressure treatments were enrolled in 21 of 24 studies (three did not provide this information). There was a broad range of OSA severity at baseline across studies; patients' mean baseline AHI ranged from 15 to 58 events/hour. In all studies, most patients were either overweight or obese (body mass index ranged from 29.9 to 42 kg/m^2^). None of the studies selectively focused on patients with other comorbidities. Study sample sizes ranged from 10 to 181 patients (total 1017 across studies). Study durations ranged from three weeks to nine months, with the majority of studies lasting three months or less. Two trials were rated quality A, 12 were rated quality B and ten rated quality C. Primary methodological concerns included small sample sizes without statistical power calculations, incomplete data reporting, short follow-up durations and high dropout rates. Based primarily on the eligibility criteria and baseline characteristics of the trial, the outcomes are applicable mainly to newly diagnosed (previously untreated) OSA patients with AHI > 15 events/hour and body mass index > 30 kg/m^2^. Outcome-specific tables summarizing the trials and their results can be found Table 2 (compliance), Table 3 (AHI), Table 4 (ESS), Table 5 (arousal index), Table 6 (minimum O_2 _saturation), Table 7 (sleep efficiency), Table 8 (rapid eye movement (REM) sleep), Table 9 (stage 3 or 4 sleep) and Table 10 (quality of life and functional outcomes).

**Table 1:** Randomized controlled trials of APAP versus CPAP: baseline study characteristics

Study PMID	Study design	Mean age (years)	Male, %	Mean body mass index (kg/m^2^)	Baseline AHI (SD)	Previous CPAP?	Funding	Quality issues
d'Ortho 2000 [32] 11035671	XO	57	88	32	58 (6)	No	Industry/Non-industry combined	-
Damjanovic 2009 [10] 19129293	PL	57	78	31.1	44 (25)	No (new Dx)	nd	-
Fietze 2007 [11] 17337881	PL	54	95	30.9	42 (26)	No (new Dx)	Industry	Incomplete reporting; patient selection unclear
Galetke 2008 [12] 17148931	XO	56	80	29.3	33 (19)	No (new Dx)	nd	Incomplete reporting; small sample and no power calculation
Hudgel 2000 [18] 10947032	XO	46	54	42	30 (25)	No	nd	Incomplete reporting; 35% dropout
Hukins 2004 [19] 15683142	XO	50	87	35.2	50 (25)	No	Industry	-
Hussain 2004 [20] 15072173	XO	45	90	35.9	47 (36)	No	Industry	Patient recruitment method unclear; small sample and no power calculation
Konermann 1998 [31] 9515848	PL	54	88	32.1	38 (14)	nd	nd	-
Marrone 2004 [21] 15165530	XO	53	95	32.9	68 (12)	No	nd	Small sample and no power calculation
Massie 2003 [22] 12406840	XO	49	82	32	nd	No (new Dx, implied)	Industry	Incomplete reporting
Meurice 2007 [13] 17638595	PL	55	nd	30.8	55 (10)	No (no Tx)	nd	Patient recruitment unclear
Nolan 2007 [14] 17326544	XO	53	90	29.9	15 (8)	No (new Dx)	Non-industry	-
Noseda 2004 [23] 15249439	XO	49	96	32.3	nd	No	nd	-
Nussbaumer 2006 [15] 16537862	XO	49	90	31.1	41 (20)	nd	Industry	-
Patruno 2007 [16] 17494789	PL	48	81	36.5	46 (14)	No (new Dx)	Non-industry	Incomplete reporting
Planes 2003 [29] 12683473	PL	54	77	32.4	59 (17)	No (new Dx)	Industry (unclear)	Patient recruitment unclear
Randerath 2001 [26] 11254519	XO	55	87	32.4	35 (26)	nd	Industry	-
Resta 2004 [24] 15679008	PL	33	90	36.7	47 (11)	No (no Tx)	nd	Incomplete reporting
Senn 2003 [25] 14525804	XO	53	79	33.3	46 (23)	No	nd	-
Sériès 1997 [27] 9341056	PL	36-65 (range)	nd	36.4	44 (20)	No (no Tx)	Industry	Potential selection bias
Teschler 2000 [28] 10885414	XO	52	100	33.8	53 (26)	No (new Dx)	nd	Incomplete reporting; small sample
To 2008 [17] 18197915	XO	46	nd	28.7	54 (nd)	No (new Dx)	nd	-
Vennelle 2010 [30] 20175411	XO	50	77	34.5	33 (18)	No (new Dx)	nd	-
West 2006 [33] 16254055	PL	46.5^a^	85	nd	nd	No	Industry	-

APAP: auto-titrating CPAP; CPAP: continuous positive airway pressure; nd: no data; new Dx: a new diagnosis of sleep apnea; no Tx: no prior treatment; PL: parallel; XO: cross-over. ^a^Median of the CPAP arm.

**Table 2:** Compliance (mean hours/night) in randomized controlled trials of APAP versus CPAP

Study PMID	Baseline AHI (SD) [Eligibility]	Baseline ESS (SD)	Duration (design)	Interventions	Number analyzed	Final	Change (final)	Net difference or difference	*P *	Dropout (%)	Study quality
d'Ortho 2000 [32] 11035671	58 (6) [≥10]	12.7 (5.3)	2 months (XO)	APAP	25	4.1	-0.6	-1.5, 0.32^a^	0.20	0	B
				CPAP	25	4.7					
Damjanovic 2009 [10] 19129293	44 (25) [≥15]	8.8 (5.2)	3 months (PL)	APAP	46	5.4	0	-0.7, 0.7	nd	8	B
				CPAP	46	5.4					
			9 months (PL)	APAP	34	5.2	0.1	-0.9, 1.1	nd	22	
				CPAP	44	5.1					
Fietze 2007 [11] 17337881	42 (26) [≥10]	nd	1.5 months (PL)	APAP	20	5.0	0.8	nd	NS	0	C
				CPAP	21	4.2					
Galetke 2008 [12] 17148931	33 (19) [> 10]	10.3 (5.7)	2 months (XO)	APAP	20	6.37	-0.01	-0.82, 0.8	nd	nd	C
				CPAP	20	6.38					
Hudgel 2000 [18] 10947032	30 (25) [nd]	16.0 (5.0)	3 months (XO)	APAP	14	6.0	0.5	0.02, 0.98^a^	< 0.04	35	C
				CPAP	19	5.5					
Hukins 2004 [19] 15683142	56 (nd)	12.5 (nd)	1 to 2 months (XO)	APAP	46	5.05	0.19	-0.06, 0.44^a^	0.14	16	B
				CPAP	46	4.86					
Hussain 2004 [20] 15072173	47 (36) [> 15]	11.1 (6.4)	1 month (XO)	APAP	10	4.3	0.6	-0.84, 2.04	nd	0	C
				CPAP	10	3.7					
Konermann 1998 [31] 9515848	38 (14) [> 20]	nd	3 to 6 months (PL)	APAP	25	6.5	0.8	0.19, 1.41^a^	< 0.01	4	B
				CPAP	23	5.7					
Marrone 2004 [21] 15165530	68 (12) [30]	16.3 (5.0)	1 month (XO)	APAP	22	4.9	0.5	-0.26, 1.26	nd	0	C
				CPAP	22	4.4					
Massie 2003 [22] 12406840	nd [≥15]	nd	1.5 months (XO)	APAP	44	5.1	0.58	0.18, 0.99^a^	0.005	4	B
				CPAP	44	4.52					
Meurice 2007 [13] 17638595	55 (10) [nd]	11.8 (4.9)	3 months (PL)	APAP (AutoSet)	15	6.0	-0.1	-0.79, 0.59	nd	15	B
				CPAP	14	6.1					
			6 months (PL)	APAP (AutoSet)	15	6.1	-0.4	-1.28, 0.48	nd	15	
				CPAP	14	6.5					
Nolan 2007 [14] 17326544	15 (8) [≥5]	12.3 (4.0)	2 months (XO)	APAP	29	4.9	0	nd	0.94	15	B
				CPAP	29	4.9					
Noseda 2004 [23] 15249439	51 (25) [> 20]	10.7 (2.4)	2 months (XO)	APAP	24	5.3	-0.2	-0.89, 0.49	nd	11	B
				CPAP	24	5.5					
Nussbaumer 2006 [15] 16537862	41 (20) [> 10]	12.7 (3.3)	1 month (XO)	APAP	30	5.1	0.3	-0.29, 0.89	nd	12	B
				CPAP	30	4.8					
Patruno 2007 [16] 17494789	46 (14) [> 20]	15 (2.7)	3 months (PL)	APAP	15	6.2	0.2	-0.25, 0.65	nd	23	C
				CPAP	16	6.0					
Planes 2003 [29] 12683473	59 (17) [≥30]	15.1 (3.9)	2 months (PL)	APAP	16	4.5	-0.8	nd	NS	14	C
				CPAP	14	5.3					
Randerath 2001 [26] 11254519	35 (26) [≥10]	11.1 (5.1)	1.5 months (XO)	APAP	46	5.26	0	-0.44, 0.44	nd	12	B
				CPAP	46	5.26					
Resta 2004 [24] 15679008	47 (11) [> 30]	13.9 (3.2)	1 month (PL)	APAP	10	5.2	-0.1	-1.12, 0.92	nd	0	C
				CPAP	10	5.3					
Senn 2003 [25] 14525804	46 (23) [> 10]	14.2 (3.8)	1 month (XO)	APAP (Autoset T)	29	5.5	-0.1	nd	NS	7	B
				APAP (AutoAdjust)	29	5.5	-0.1	nd	NS		
				CPAP	29	5.6					
Sériès 1997 [27] 9341056	44 (20) [nd]	15.5 (4.5)	0.75 months (PL)	APAP^b^	12	nd^c^	-	-	NS	0	C
				CPAP	12	nd					
				APAP^d^	12	nd^c^	-	-	NS		
				CPAP	12	nd					
Vennelle 2010 [30] 20175411	33 (18) [≥15]	14 (3)	6 weeks (XO)	APAP	181	4.2	0.2	0.003, 0.397	0.047	9.5	A
				CPAP	181	4.0					
Teschler 2000 [28] 10885414	53 (26) [> 20]	nd	2 months (XO)	APAP	10	6.3	0.2	-0.7, 1.1	nd	nd	C
				CPAP	10	6.1					
To 2008 [17] 18197915	54 (nd) [> 30]	13.4 (nd)	1 month (XO)	APAP	41	4.3	0.5	0.02, 0.98	0.04	5	B
				CPAP	41	3.8					
			2 months (XO)	APAP	41	4.4	0.7	0.17, 1.23^a^	0.01		
				CPAP	41	3.7					
West 2006 [33] 16254055	33^e ^(nd) [> 10]	16.5^f ^(nd)	1 month (PL)	APAP	29	5.3			0.27	6.1	A
				CPAP (auto)	31	4.3					
				CPAP (algo)	32	4.7					
			6 months (PL)	APAP	28	5.5			0.23		
				CPAP (auto)	31	4.9					
				CPAP (algo)	27	4.0				9.2	

AHI: apnea-hypopnea index (events/hour); algo: fixed pressure CPAP determined by algorithm; APAP: auto-titrating CPAP; auto: fixed pressure CPAP determined by pressures from 1 week of APAP; CPAP: continuous positive airway pressure; ESS: Epworth Sleepiness Scale; nd: no data; NS: non-significant; PL: parallel design; XO: cross-over design. ^a^Estimated from reported *P *value. ^b^Estimated effective pressure. ^c^Directions of changes not reported in the study. ^d^Measured effective pressure. ^e^> 4% SaO_2 _dip rate per hour in algorithm-determined CPAP arm, median. ^f^Median in algorithm-determined CPAP arm.

**Table 3:** AHI (events/hour) in randomized controlled trials of APAP versus CPAP

Study PMID	Baseline AHI (SD) [eligibility]	Baseline ESS (SD)	Duration (design)	Interventions	Number analyzed	Baseline (SD)	Change (final)	Net difference or difference	95% CI^a^	*P *	Dropout (%)	Study quality
d'Ortho 2000 [32] 11035671	58 (6) [≥10]	12.7 (5.3)	2 months (XO)	APAP	25	57.8 (5.8)	-47.2	0.9	nd	NS	0	B
				CPAP	25	57.8 (5.8)	-48.1					
Damjanovic 2009 [10] 19129293	44 (25) [≥15]	8.8 (5.2)	3 months (PL)	APAP	46	41.8 (23.7)	-37.0	1.8	-7.14 to 10.74	nd	8	B
				CPAP	46	45.5 (24.4)	-38.8					
			9 months (PL)	APAP	34	41.8 (23.7)	-38.2	1.9	-6.86 to 10.66	nd	22	
				CPAP	44	45.5 (24.4)	-40.1					
Fietze 2007 [11] 17337881	42 (26) [≥10]	nd	1.5 months (PL)	APAP	20	43.3 (30.2)	-38.9	0.5	-1.19 to 2.19	nd	0	C
				CPAP	21	40.4 (26.1)	-36.5					
Galetke 2008 [12] 17148931	33 (19) [> 10]	10.3 (5.7)	2 months (XO)	APAP	20	32.9 (19.1)	-27.3	1.0	-0.45 to 2.45	nd	nd	C
				CPAP	20	32.9 (19.1)	-28.3					
Hussain 2004 [20] 15072173	47 (36) [> 15]	11.1 (6.4)	1 month (XO)	APAP	10	47.2 (35.6)	-34.1	3.5	-1.02 to 8.02	nd	0	C
				CPAP	10	47.2 (35.6)	-37.6					
Konermann 1998 [31] 9515848	38 (14) [> 20]	nd	3 to 6 months (PL)	APAP	25	35.5 (9.6)	-33.1	1.6	nd	NS	4	B
				CPAP	23	38.3 (13.9)	-34.7					
Massie 2003 [22] 12406840	nd [≥15]	nd	1.5 months (XO)	APAP	44	nd	nd	-1.1	-2.89 to 0.69	nd	4	B
				CPAP	44	nd	nd					
Meurice 2007 [13] 17638595	55 (10) [nd]	11.8 (4.9)	6 months (PL)	APAP (AutoSet)	15	53.4 (15.1)	-51.1	2.6	-8.88 to 14.08	nd	15	B
				CPAP	14	56.1 (21.4)	-53.7					
Nolan 2007 [14] 17326544	15 (8) [≥5)	12.3 (4.0)	2 months (XO)	APAP	29	14.7 (8)	-12.0	-0.8	-1.89 to 0.29^b^	0.15	15	B
				CPAP	29	14.7 (8)	-11.2					
Nussbaumer 2006 [15] 16537862	41 (20) [> 10]	12.7 (3.3)	1 month (XO)	APAP	30	41.1 (19.7)	-36.5	-0.8	-1.7 to 3.3^c^	nd	12	B
				CPAP	30	41.2 (19.7)	-35.7					
Patruno 2007 [16] 17494789	46 (14) [> 20]	15 (2.7)	3 months (PL)	APAP	15	47.3 (14.7)	-41.3	2.7	-7.01 to 12.41	nd	23	C
				CPAP	16	46.0 (14.6)	-44.0					
Planes 2003 [29] 12683473	59 (17) [≥30]	15.1 (3.9)	2 months (PL)	APAP	16	57.5 (16.5)	-49.9	0.7	-10.06 to 11.46	nd	14	C
				CPAP	14	61.0 (17.4)	-50.6					
Randerath 2001 [26] 11254519	35 (26) [≥10]	11.1 (5.1)	1.5 months (XO)	APAP	52	35.1 (26)	-30.1	0.7	-0.88 to 2.28	nd	12	B
				CPAP	52	35.1 (26)	-30.8					
Resta 2004 [24] 15679008	47 (11) [> 30]	13.9 (3.2)	1 month (PL)	APAP	10	48.0 (14.3)	-39.7	-2.8	-12.96 to 7.36	nd	0	C
				CPAP	10	45.3 (10.7)	-36.9					
Senn 2003 [25] Switzerland 14525804	46 (23) [> 10]	14.2 (3.8)	1 month (XO)	APAP (Autoset T)	29	45.8 (22.6)	-39.8	0.7	-1.26 to 2.66	nd	7	B
				APAP (AutoAdjust)	29	45.8 (22.6)	-38.1	2.4	-0.34 to 5.14	nd		
				CPAP	29	45.8 (22.6)	-40.5					
Sériès 1997 [27] 9341056	44 (20) [nd]	15.5 (4.5)	0.75 months (PL)	APAP^d^	12	61.5 (27.9)	nd^e^	-	-	NS	0	C
				CPAP	12	50.1 (14.5)	nd					
				APAP^f^	12	46.8 (22.3)	nd^e^	-	-	NS		
				CPAP	12	50.1 (14.5)	nd					

AHI: apnea-hypopnea index (events/hour); APAP: auto-titrating CPAP; CPAP: continuous positive airway pressure; ESS: Epworth Sleepiness Scale; nd: no data; NS: non-significant; PL: parallel design; XO: cross-over design. ^a^Estimated from reported data. ^b^Estimated from reported *P *value. ^c^Actual reported data. ^d^Estimated effective pressure. ^e^Directions of changes not reported in the study. ^f^Measured effective pressure.

**Table 4:** ESS in randomized controlled trials of APAP versus CPAP

Study PMID	Baseline AHI (SD) [eligibility]	Baseline ESS (SD)	Duration (design)	Interventions	Number analyzed	Baseline (SD)	Change (final)	Net difference or difference	95% CI^a^	*P *	Dropout (%)	Study quality
d'Ortho 2000 [32] 11035671	58 (6) [≥10]	12.7 (5.3)	2 months (XO)	APAP	25	12.7 (5.3)	-3.4	0.1	nd	NS	0	B
				CPAP	25	12.7 (5.3)	-3.5					
Damjanovic 2009 [10] 19129293	44 (25) [≥15]	8.8 (5.2)	3 months (PL)	APAP	46	8.5 (5.4)	-2.6	-0.3	-2.32 to 1.72	nd	8	B
				CPAP	46	9.3 (4.8)	-2.3					
			9 months (PL)	APAP	34	8.5 (5.4)	-2.6	0.1	-1.92 to 2.12	nd	22	
				CPAP	44	9.3 (4.8)	-2.7					
Fietze 2007 [11] 17337881	42 (26) [≥10]	nd	1.5 months (PL)	APAP	20	nd	nd	nd	nd	NS	0	C
				CPAP	21	nd	nd					
Galetke 2008 [12] 17148931	33 (19) [> 10]	10.3 (5.7)	2 months (XO)	APAP	20	10.3 (5.7)	-5.4	-1.7	-3.76 to 0.36	nd	0	C
				CPAP	20	10.3 (5.7)	-3.7					
Hudgel 2000 [18] 10947032	30 (25) [nd]	16.0 (5.0)	3 months (XO)	APAP	39	16.0 (5.0)	-7.0	1	-0.96 to 2.96	nd	35	C
				CPAP	39	16.0 (5.0)	-8.0					
Hussain 2004 [20] 15072173	47 (36) [> 15]	11.1 (6.4)	1 months (XO)	APAP	10	11.1 (6.4)	-3.1	1.4	-2.2 to 5.0	nd	0	C
				CPAP	10	11.1 (6.4)	-4.5					
Hukins 2004 [19] 15683142	56 (nd) [≥5]	12.5 (nd)	1 to 2 months (XO)	APAP	46	12.5 (nd)	-4.5	-0.2	nd	NS	16	B
				CPAP	46	12.5 (nd)	-4.3					
Marrone 2004 [21] 15165530	68 (12) [30]	16.3 (5.0)	1 month (XO)	APAP	22	16.3 (5.0)	-12.4	-1	-2.4 to 0.4	nd	0	C
				CPAP	22	16.3 (5.0)	-11.4					
Massie 2003 [22] 12406840	nd [≥15]	nd	1.5 months (XO)	APAP	44	nd	nd	-1	-2.06 to 0.06^b^	0.065	4	B
				CPAP	44	nd	nd					
Meurice 2007 [13] 17638595	55 (10) [nd]	11.8 (4.9)	3 months (PL)	APAP (AutoSet)	15	12.9 (4.3)	-9.1	-3.3	-6.68 to 0.08	NS	15	B
				CPAP	14	10.6 (5.2)	-5.8					
			6 months (PL)	APAP (AutoSet)	15	12.9 (4.3)	-7.7	-3.0	-6.44 to 0.44	NS	15	
				CPAP	14	10.6 (5.2)	-4.7					
Nolan 2007 [14] 17326544	15 (8) [≥5)	12.3 (4.0)	2 months (XO)	APAP	29	12.3 (4.0)	-3.7	0.9	-0.99 to 2.79^b^	0.35	15	B
				CPAP	29	12.3 (4.0)	-4.6					
Noseda 2004 [23] 15249439	nd [> 20]	10.7 (2.4)	2 months (XO)	APAP	24	10.7 (2.4)	nd	-1	-1.76 to -0.24^b^	< 0.01	11	B
				CPAP	24	10.7 (2.4)	nd					
Nussbaumer 2006 [15] 16537862	41 (20) [> 10]	12.7 (3.29)	1 month (XO)	APAP	30	12.7 (0.6)	-6.6	0	-1.6 to 1.1	nd	12	B
				CPAP	30	12.7 (0.6)	-6.6					
Patruno 2007 [16] 17494789	46 (14) [> 20]	15 (2.7)	3 months (PL)	APAP	15	15.8 (3.5)	nd	nd	nd	NS	23	C
				CPAP	16	14.1 (1.7)	nd					
Planes 2003 [29] 12683473	59 (17) [≥30]	15.1 (3.9)	2 months (PL)	APAP	16	15.5 (4.7)	-8.0	-0.9	-3.72 to 1.92	nd	14	
				CPAP	14	14.7 (3.9)	-7.1					
Randerath 2001 [26] 11254519	35 (26) [≥10]	11.1 (5.1)	1.5 months (XO)	APAP	52	11.1 (5.1)	-3.3	-1	-2.26 to 0.26	nd	12	B
				CPAP	52	11.1 (5.1)	-2.3					
Resta 2004 [24] 15679008	47 (11) [> 30]	13.9 (3.2)	1 month (PL)	APAP	10	15.7 (5.1)	-10.5	-2.6	-5.84 to 0.64	nd	0	C
				CPAP	10	12.0 (3.2)	-7.9					
Senn 2003 [25] Switzerland 14525804	46 (23) [> 10]	14.2 (3.77)	1 month (XO)	APAP (Autoset T)	29	14.2 (3.77)	-5.2	0.8	-0.49 to 2.09	nd	7	B
				APAP (AutoAdjust)	29	14.2 (3.77)	-6.2	-0.2	-1.68 to 1.28	nd		
				CPAP	29	14.2 (3.77)	-6.0					
Sériès 1997 [27] 9341056	44 (20) [nd]	15.5 (4.5)	0.75 months (PL)	APAP^c^	12	17.0 (4.1)	-9.1	-0.8	-4.28 to 2.69	nd	0	C
				CPAP	12	16.1 (4.5)	-8.3					
				APAP^d^	12	13.5 (4.7)	-6.5	1.8	-1.78 to 5.38	nd		
				CPAP	12	16.1 (4.5)	-8.3					
To 2008 [17] 18197915	54 (nd) [> 30]	13.4 (nd)	1 month (XO)	APAP	41	13.4 (5.76)	-4.9	0.3	-1.46 to 2.06	nd	5	B
				CPAP	41	13.4 (5.76)	-5.2					
			2 months (XO)	APAP	41	13.4 (5.76)	-4.9	0	-1.76 to 1.76	nd		
				CPAP	41	13.4 (5.76)	-4.9					
Vennelle 2010 [30] 20175411	33 (18) [≥15]	14 (3)	6 weeks (XO)	APAP	181	14 (3)	-4.5	-0.5	-0.95 to -0.05	0.031	9.5	A
				CPAP	181	14 (3)	-4					
West 2006 [33]	33^e ^(nd) [> 10]	16.5^f^(nd)	1 month (PL)	APAP	29	16 (median)	7 (final)			0.9	6.1	A
				CPAP (auto)	31	17 (median)	7 (final)					
				CPAP (algo)	32	16.5 (median)	6 (final)					
			6 months (PL)	APAP	28	16 (median)	6 (final)			0.8	9.2	
				CPAP (auto)	31	17 (median)	5 (final)					
				CPAP (algo)	27	16.5 (median)	5 (final)					

AHI: apnea-hypopnea index (events/hour); algo: fixed pressure CPAP determined by algorithm; APAP: auto-titrating CPAP; auto: fixed pressure CPAP determined by pressures from 1 week of APAP; CPAP: continuous positive airway pressure; ESS: Epworth Sleepiness Scale; nd: no data; NS: non-significant; PL: parallel design; XO: cross-over design. ^a^Estimated from reported data. ^b^Estimated from reported P value. ^c^Estimated effective pressure. ^d^Measured effective pressure. ^e^> 4% SaO_2 _dip rate per hour in algorithm-determined CPAP arm. ^f^Median in algorithm-determined CPAP arm.

**Table 5:** Arousal index (events/hour) in randomized controlled trials of APAP versus CPAP

Study PMID	Baseline AHI (SD) [eligibility]	Baseline ESS (SD)	Duration (design)	Interventions	Number analyzed	Baseline (SD)	Change (final)	Net difference or difference	95% CI^a^	*P *	Dropout (%)	Study quality
d'Ortho 2000 [32] 11035671	58 (6) [≥10]	12.7 (5.3)	2 months (XO)	APAP	25	45.6 (25.8)	-30.1	1.8	nd	NS	0	B
				CPAP	25	45.6 (25.8)	-31.9					
Damjanovic 2009 [10] 19129293	44 (25) [≥15]	8.8 (5.18)	3 months (PL)	APAP	46	30.6 (22.4)	-18.3	-0.2	-7.92 to 7.52	nd	8	B
				CPAP	46	34.5 (21.0)	-18.1					
			9 months (PL)	APAP	34	30.6 (22.4)	-17.7	3.6	-4.09 to 11.29	nd	22	
				CPAP	44	34.5 (21.0)	-21.3					
Galetke 2008 [12] 17148931	33 (19) [> 10]	10.3 (5.7)	2 months (XO)	APAP	20	17.6 (9.2)	-4.0	1.0	-2.52 to 4.52	nd	nd	C
				CPAP	20	17.6 (9.2)	-5.0					
Hussain 2004 [20] 15072173	47 (36) [> 15]	11.1 (6.4)	1 month (XO)	APAP	10	17.3 (17.7)	-11.4	1.0	-2.5 to 4.5	nd	0	C
				CPAP	10	17.3 (17.7)	-12.4					
Konermann 1998 [31] 9515848	38 (14) [> 20}	nd	3 to 6 months (PL)	APAP	25	16.9 (10.5)	-9.5	-3.3	-6.6 to 0^b^	< 0.05	4	B
				CPAP	23	13.3 (13.3)	-6.2					
Nolan 2007 [14] 17326544	15 (8) [≥5)	12.3 (4)	2 months (XO)	APAP	29	16.0 (14.0)	-14.0	-3.0	-5.7 to -0.29^b^	0.03	15	B
				CPAP	29	16.0 (14.0)	-11.0					
Planes 2003 [29] 12683473	59 (17) [≥30]	15.1 (3.9)	2 months (PL)	APAP	16	44.4 (19.1)		3.3	-7.14 to 12.74	nd	14	C
				CPAP	14	48.5 (14.2)						
Randerath 2001 [26] 11254519	35 (26) [≥10]	11.1 (5.1)	1.5 months (XO)	APAP	52	34 (21.7)	-23.1	-1.7	-3.7 to 0.3	nd	12	B
				CPAP	52	34 (21.7)	-21.4					
Resta 2004 [24] 15679008	47 (11) [> 30]	13.9 (3.2)	1 month (PL)	APAP	10	43.1 (11.9)	-35.7	0.1	-8.29 to 8.49	nd	0	C
				CPAP	10	43.1 (9.1)	-35.8					
Sériès 1997 [27] 9341056	44 (20) [nd]	15.5 (4.5)	0.75 months (PL)	APAP^c^	12	nd	nd	-	-	NS	0	C
				CPAP	12	nd	nd					
				APAP^d^	12	nd	nd	-	-	NS		
				CPAP	12	nd	nd					

AHI: apnea-hypopnea index (events/hour); APAP: auto-titrating CPAP; CPAP: continuous positive airway pressure; ESS: Epworth Sleepiness Scale; nd: no data; NS: non-significant; PL: parallel design; XO: cross-over design. ^a^Estimated from reported data. ^b^Estimated from reported *P *value. ^c^Estimated effective pressure. ^d^Measured effective pressure.

**Table 6:** Minimum O_2 _saturation (%) in randomized controlled trials of APAP versus CPAP

Study PMID	Baseline AHI (SD) [eligibility]	Baseline ESS (SD)	Duration (design)	Interventions	Number analyzed	Baseline (SD)	Change (final)	Net difference or difference	95% CI^a^	*P*	Dropout (%)	Study quality
d'Ortho 2000 [32] 11035671	58 (6) [≥10]	12.7 (5.3)	2 months (XO)	APAP	25	66.5 (13.6)	85.2	-1.4	nd	nd	0	B
				CPAP	25	66.5 (13.6)	86.6					
Galetke 2008 [12] 17148931	33 (19) [> 10]	10.3 (5.7)	2 months (XO)	APAP	20	77.8 (8.4)	8.7	-1.8	-3.8 to 0.2	nd	nd	C
				CPAP	20	77.8 (8.4)	10.5					
Hussain 2004 [20] 15072173	47 (36) [> 15]	11.1 (6.4)	1 month (XO)	APAP	10	67.8 (12.5)	14	-3.9	-7.3 to -0.5	nd	0	C
				CPAP	10	67.8 (12.5)	17.9					
Konermann 1998 [31] 9515848	38 (14) [> 20}	nd	3 to 6 months (PL)	APAP	25	76.5 (12.4)	90.3	1.1	nd	NS	4	B
				CPAP	23	74.5 (10.7)	87.2					
Meurice 2007 [13] 17638595	55 (10) [nd]	11.8 (4.9)	6 months (PL)	APAP (AutoSet)	15	82.1 (12.8)	0.2	-2.9	-7.1 to 7.5	nd	15	B
				CPAP	14	82.3 (9.9)	0.5					
Nolan 2007 [14] 17326544	15 (8) [≥5)	12.3 (4)	2 months (XO)	APAP	29	79 (11.5)	8.5	4.8	-7.4 to 17.0^b^	0.44	15	B
				CPAP	29	79 (11.5)	3.7					
Patruno 2007 [16] 17494789	46 (14) [> 20]	15 (2.7)	3 months (PL)	APAP	15	71.7 (10.6)	16.4	-4.4	-11.8 to 3.0	nd	23	C
				CPAP	16	70.0 (11.7)	20.8					
Randerath 2001 [26] 11254519	35 (26) [≥10]	11.1 (5.1)	1.5 months (XO)	APAP	52	81 (8.0)	7.0	-1.0	-2.1 to 0.1	nd	12	B
				CPAP	52	81 (8.0)	8.0					
Resta 2004 [24] 15679008	47 (11) [> 30]	13.9 (3.2)	1 month (PL)	APAP	10	72.4 (10.5)	15.9	0.9	-7.4 to 9.2	nd	0	C
				CPAP	10	74.1 (10.8)	15.0					

AHI: apnea-hypopnea index (events/hour); APAP: auto-titrating CPAP; CPAP: continuous positive airway pressure; ESS: Epworth Sleepiness Scale; nd: no data; NS: non-significant; PL: parallel design; XO: cross-over design. ^a^Estimated from reported data. ^b^Estimated from reported *P *value.

**Table 7:** Sleep efficiency (%) in randomized controlled trials of APAP versus CPAP

Study PMID	Baseline AHI (SD) [eligibility]	Baseline ESS (SD)	Duration (design)	Interventions	Number analyzed	Baseline (SD)	Change (final)	Net difference or difference	95% CI	*P *	Dropout (%)	Study quality
Konermann 1998 [31] 9515848	38 (14) [> 20}	nd	3 to 6 months (PL)	APAP	25	94.5 (5.4)	2	6	nd	NS	4	B
				CPAP	23	89.2 (13.7)	-4					
Nolan 2007 [14] 17326544	15 (8) [≥5)	12.3 (4)	2 months (PL)	APAP	29	79 (9)	4	-1	-4.3 to 2.3^a^	0.39	15	B
				CPAP	29	79 (9)	5					
Resta 2004 [24] 15679008	47 (11) [> 30]	13.9 (3.2)	1 month (PL)	APAP	10	86.9 (8)	-0.5	-2.5	nd	nd	0	C
				CPAP	10	84.2 (4.9)	2					

AHI: apnea-hypopnea index (events/hour); APAP: auto-titrating CPAP; CPAP: continuous positive airway pressure; ESS: Epworth Sleepiness Scale; nd: no data; NS: non-significant; PL = parallel design. ^a^Estimated from reported data.

**Table 8:** Rapid eye movement sleep (%) in randomized controlled trials of APAP versus CPAP

Study PMID	Baseline AHI (SD) [eligibility]	Baseline ESS (SD)	Duration (design)	Interventions	Number analyzed	Baseline (SD)	Change (final)	Net difference or difference	95% CI^a^	*P *	Dropout (%)	Study quality
d'Ortho 2000 [32] 11035671	58 (6) [≥10]	12.7 (5.3)	2 months (XO)	APAP	25	16 (5)	5	-1	nd	nd	0	B
				CPAP	25	16 (5)	6					
Hussain 2004 [20] 15072173	47 (36) [> 15]	11.1 (6.4)	1 month (XO)	APAP	10	15 (7.0)	4.0	-1	-4.72 to 2.72	nd	0	C
				CPAP	10	17.6 (5.1)	2.0					
Konermann 1998 [31] 9515848	38 (14) [> 20]	nd	3 to 6 months (PL)	APAP	25	8.2 (8.1)	11.9	7.1	nd	NS	4	B
				CPAP	23	5.4 (6.0)	4.8					
Meurice 2007 [13] 17638595	55 (10) [nd]	11.8 (4.9)	6 months (PL)	APAP (AutoSet)	15	18.9 (6.6)	-2.4	-2.9	-7.49 to 1.69	nd	15	B
				CPAP	14	19.1 (5.9)	0.5					
Nolan 2007 [14] 17326544	15 (8) [≥5)	12.3 (4)	2 months (XO)	APAP	29	17.6 (5.1)	-0.5	-2.5	-5.11 to 0.11^b^	0.06	15	B
				CPAP	29	17.6 (5.1)	2.0					
Planes 2003 [29] 12683473	59 (17) [≥30]	15.1 (3.9)	2 months (PL)	APAP	16	12.4 (7.0)	4.2	0.5	-5.44 to 6.44	nd	14	C
				CPAP	14	13.7 (9.3)	3.7					
Randerath 2001 [26] 11254519	35 (26) [≥10]	11.1 (5.1)	1.5 months (XO)	APAP	52	11 (8)	6.0	1	-0.63 to 2.63	nd	12	B
				CPAP	52	11 (8)	5.0					
Resta 2004 [24] 15679008	47 (11) [> 30]	13.9 (3.2)	1 month (PL)	APAP	10	15.0 (8.1)	6.7	-2	-10.31 to 6.31	nd	0	C
				CPAP	10	15.9 (4.2)	8.7					
Sériès 1997 [27] 9341056	44 (20) [nd]	15.5 (4.5)	0.75 months (PL)	APAP^c^	12	nd	nd^d^	-	-	NS	0	C
				CPAP	12	nd	nd					
				APAP^e^	12	nd	nd^d^	-	-	NS		
				CPAP	12	nd	nd					

AHI: apnea-hypopnea index (events/hour); APAP: auto-titrating CPAP; CPAP: continuous positive airway pressure; ESS: Epworth Sleepiness Scale; nd: no data; NS: non-significant; PL: parallel design; XO: cross-over design. ^a^Estimated from reported data. ^b^Estimated from reported *P *value. ^c^Estimated effective pressure. ^d^Directions of changes not reported in the study. ^e^Measured effective pressure.

**Table 9:** Stage 3 or 4 sleep (%) in randomized controlled trials of APAP versus CPAP

Study PMID	Baseline AHI (SD) [eligibility]	Baseline ESS (SD)	Duration (design)	Interventions	Number analyzed	Baseline (SD)	Change (final)	Net difference or difference	95% CI^a^	*P *	Dropout (%)	Study quality
d'Ortho 2000 [32] 11035671	58 (6) [≥10]	12.7 (5.3)	2 months (XO)	APAP	25	44 min (36)	43	-9 min	nd	NS	0	B
				CPAP	25	44 min (36)	52					
Hussain 2004 [20] 15072173	47 (36) [> 15]	11.1 (6.4)	1 month (XO)	APAP	10	14 (25)	-4.0	-8	-18.32 to 2.32	nd	0	C
				CPAP	10	14 (25)	4.0					
Konermann 1998 [31] 9515848	38 (14) [> 20]	nd	3 to 6 months (PL)	APAP	25	13.2 (12.2)	14	7.8	1.8 to 13.7^b^	< 0.01	4	B
				CPAP	23	11.4 (10.4)	6.2					
Meurice 2007 [13] 17638595	55 (10) [nd]	11.8 (4.9)	6 months (PL)	APAP (AutoSet)	15	25.5 (14.7)	-4.4	-5.2	-13.51 to 3.11	nd	15	B
				CPAP	14	17.1 (7)	0.8					
Nolan 2007 [14] 17326544	15 (8) [≥5)	12.3 (4)	2 months (XO)	APAP	29	13.7 (7.8)	1.3	0.3	-3.29 to 3.89^b^	0.87	15	B
				CPAP	29	13.7 (7.8)	1.0					
Randerath 2001 [26] 11254519	35 (26) [≥10]	11.1 (5.1)	1.5 months (XO)	APAP	52	14 (11)	0	-1	-3.45 to 1.45	NS	12	B
				CPAP	52	14 (11)	1.0					
Resta 2004 [24] 15679008	47 (11) [> 30]	13.9 (3.2)	1 month (PL)	APAP	10	19.8 (10.9)	14	7.3	-2.35 to 16.95	nd	0	C
				CPAP	10	22.8 (12.5)	6.7					
Sériès 1997 [27] 9341056	44 (20) [nd]	15.5 (4.5)	0.75 months (PL)	APAP^c^	12	nd	nd^d^	-	-	NS	0	C
				CPAP	12	nd	nd					
				APAP^e^	12	nd	nd^d^	-	-	NS		
				CPAP	12	nd	nd					

AHI: apnea-hypopnea index (events/hour); APAP: auto-titrating CPAP; CPAP; continuous positive airway pressure; ESS: Epworth Sleepiness Scale; nd: no data; NS: non-significant; PL: parallel design; XO: cross-over design. ^a^Estimated from reported data. ^b^Estimated from reported *P *value. ^c^Estimated effective pressure. ^d^Directions of changes not reported in the study. ^e^Measured effective pressure.

**Table 10:** Quality of life and functional outcomes in randomized controlled trials of APAP versus CPAP

Study PMID	Baseline AHI (SD) [eligibility]	Baseline ESS (SD)	Interventions	Number analyzed	Duration (design)	Outcome	Favors	If significant difference					Dropout (%)	Study quality
										
								Net difference	95% CI	Test range		*P *		
										"Worst"	"Best"			
Fietze 2007 [11] 17337881	42 (26) [≥10]	nd	APAP	20	1.5 months (XO)	SF-36 all	0						0	C
			CPAP	21										
Hukins 2004 [19] 15683142	56 (nd) [≥5]	12.5 (nd)	APAP	46	2 months (XO)	SF-36-M	0						16	B
			CPAP	46										
						SF-36-P	0							
Massie 2003 [22] 12406840	nd [≥15]	nd	APAP	44	1.5 months (XO)	SF-36 - MH	APAP	5	0.16 to 9.8^a^	0	100	< 0.05	4	B
			CPAP	44		SF-36 - vitality	APAP	7	0.6 to 13.4^a^	0	100	< 0.05		
						SF-36 - remainder	0							
Meurice 2007 [13] 17638595	55 (10) [nd]	11.8 (4.9)	APAP (AutoSet)	15	3 months (PL)	SF-36-M	0						15	B
			CPAP	14		SF-36-P	0							
					6 months (PL)	SF-36-M	0						15	
						SF-36-P	0							
Nussbaumer 2006 [15] 16537862	41 (20) [> 10]	12.7 (3.3)	APAP	30	1 month (XO)	SF-36 all	0						12	B
			CPAP	30										
Senn 2003 [25] 14525804	46 (23) [> 10]	14.2 (3.8)	APAP (Autoset T)	29	1 month (XO)	SF-36 all	0						7	B
			APAP (AutoAdjust)	29		Vigilance (OSLER)	0							
			CPAP	29										
To 2008 [17] 18197915	54.3 (nd) [> 30]	13.4 (nd)	APAP	41	1 month (XO)	SAQLI	0						5	B
			CPAP	41	2 months (XO)	SAQLI	0							
Vennelle 2010 [30] 20175411	33 (18) [≥15]	14 (3)	APAP	181	6 weeks (XO)	SF-36-M	0						9.5	A
			CPAP	181		SF-36-P	0							
						Vigilance (OSLER)	0							
						Vigilance (Psychomotor)	0							
West 2006 [33] 16254055	33^b ^(nd) [> 10]	16.5^c ^(nd)	APAP	28	6 months (PL)	SF-36-P	0						9.2	
			CPAP (auto)	31		SF-36-M	0							
			CPAP (algo)	27		SF-36-energy, vitality	0							
						Vigilance (OSLER)	0							

AHI: apnea-hypopnea index (events/hour); algo: fixed pressure CPAP determined by algorithm; APAP: auto-titrating CPAP; auto: fixed pressure CPAP determined by pressures from 1 week of APAP; CPAP: continuous positive airway pressure; ESS: Epworth Sleepiness Scale; nd: no data; NS: non-significant; PL: parallel design; OSLER: The Oxford Sleep Resistance Test; SAQLI: Sleep Apnea Quality of Life Index; XO: cross-over design. SF-36 M: Short Form Health Survey Mental Health summary measure. SF_36 P: Short Form Health Survey Physical Health summary measure. SF-36 MH: Short Form Health Survey Mental Health scale. ^a^Estimated from reported P value. ^b^> 4% SaO2 dip rate per hour in algorithm-determined CPAP arm. ^c^Median in algorithm-determined CPAP arm.

### Objective clinical outcomes

No trial evaluated clinical outcomes, including death; cardiovascular events such as myocardial infarction, heart failure or stroke; or diabetes or depression severity.

### Compliance

**Figure 2:**
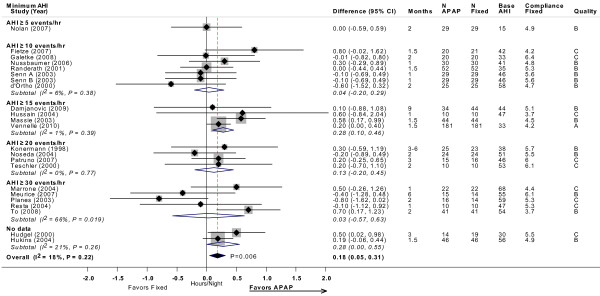
**CPAP compliance (hour/night) with APAP versus fixed CPAP: meta-analysis, with subgroup analyses by minimum AHI threshold**. Estimates and 95% CIs by study subgrouped by minimum AHI threshold used in each study. The overall random effects model meta-analysis is displayed by the black diamond, which spans the width of the 95% CI. Each subgroup meta-analysis, by AHI threshold, is shown by the open diamonds. Grey boxes are proportional to the weight of each study in the overall meta-analysis. For each meta-analysis the I^2 ^statistic and the *P *value for heterogeneity is displayed. The *P *value for the summary estimate is displayed next to the black diamond. Note that studies favoring APAP are to the right of the vertical 0 line. Base AHI: baseline apnea-hypopnea index (events/hour) in fixed CPAP group; compliance fixed: compliance (hour/night) in fixed CPAP group; fixed: fixed CPAP.

All 24 included trials reported data on compliance. The number of hours used per night was derived from machine-recorded compliance data. No statistically significant differences were observed in device usage (hours used per night) between APAP and CPAP in 20 of the trials, while four reported a significant increase in the use of APAP compared with CPAP [17,18,22,30]. Twenty-two trials provided sufficient data for meta-analysis, which showed a statistically significant difference of 11 minutes per night favoring APAP (difference = 0.18 hours; 95% CI, 0.05 to 0.31 minutes; *P *= 0.006), without statistical heterogeneity (Figure 2).

To test the *a priori *hypothesis that the relative effect on compliance may differ based on baseline severity, we performed subgroup meta-analyses stratified by minimum AHI threshold. By meta-regression, the subgroups had no significantly different effects from each other. Results were also similar in parallel and cross-over design studies.

### Apnea-hypopnea index

**Figure 3:**
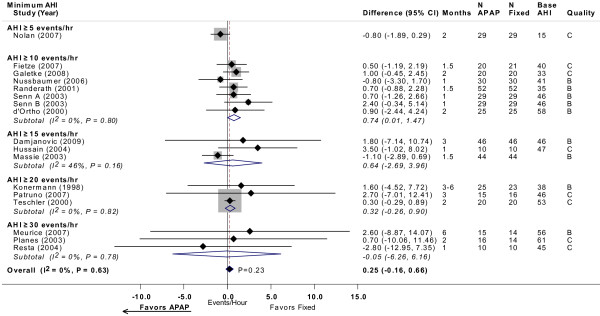
**AHI (events/hour) with APAP versus fixed CPAP: meta-analysis, with subgroup analyses by minimum AHI threshold**. See Figure 2 legend. Note that studies favoring APAP are to the left of the vertical 0 line. Senn A and Senn B were comparisons of two different APAP devices versus fixed CPAP reported in the same study. Base AHI: baseline apnea-hypopnea index (events/hour) in fixed CPAP group; fixed: fixed CPAP.

Sixteen trials provided sufficient data for analysis of residual AHI while using treatment (Figure 3) [10-16,20,22,24-26,28,29,31,32]. None of the studies reported a statistically significant difference in AHI (events/hour) between APAP and CPAP. The mean net difference in individual studies ranged from -2.8 to 3.5 events/hour, where negative values favor APAP. Meta-analysis across these studies indicated a non-significant difference between APAP and CPAP of 0.25 events/hour (95% CI, -0.16 to 0.66 events/hour; *P *= 0.23). No statistically significant heterogeneity was observed across studies, despite a broad range in the severity of OSA at baseline. Meta-regression stratified by different minimum AHI thresholds or by study design revealed no apparent differences across subgroups in the relative effects of APAP and CPAP.

### Epworth Sleepiness Scale

**Figure 4:**
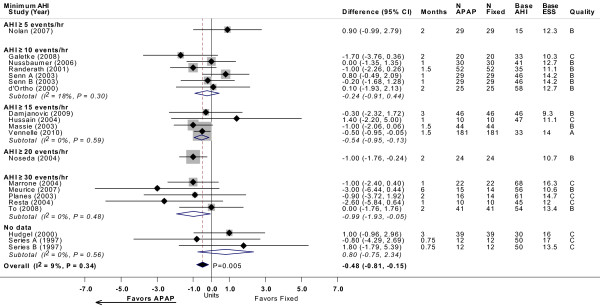
**ESS with APAP versus fixed CPAP: meta-analysis, with subgroup analyses by minimum AHI threshold**. See Figure 2 legend. Note that studies favoring APAP are to the left of the vertical 0 line. Senn A and Senn B, and Sériès A and Sériès B, were comparisons of two different APAP devices versus fixed CPAP reported in the same study, respectively. Base AHI: baseline apnea-hypopnea index (events/hour) in fixed CPAP group; base ESS: baseline Epworth Sleepiness Scale (no units) in fixed CPAP group; fixed: fixed CPAP.

Twenty-two trials reported ESS after treatment (Figure 4) [10-27,29,30,32,33]. No statistically significant differences in ESS were observed between APAP and CPAP in 20 trials, while two studies reported a significant decrease in ESS favoring APAP [23,30]. The mean net difference in ESS across all studies ranged from -3.3 to 2.0, where negative values favor less sleepiness with APAP. Eighteen trials provided sufficient data for meta-analysis, which yielded a statistically significant difference between APAP and CPAP of -0.48 (95% CI, -0.81 to -0.15; *P *= 0.005), favoring APAP. Despite the broad range of severity of OSA across studies, there was no statistically significant heterogeneity within the overall meta-analysis. Meta-regression stratified by minimum AHI threshold or by study design revealed no apparent differences across subgroups in relative effects of APAP and CPAP.

### Other sleep study measures

**Figure 5:**
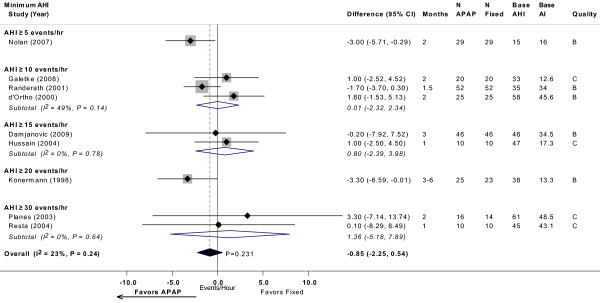
**Arousal index (events/hour) with APAP versus fixed CPAP: meta-analysis, with subgroup analyses by minimum AHI threshold**. See Figure 2 legend. Note that studies favoring APAP are to the left of the vertical 0 line. Senn A and Senn B were comparisons of two different APAP devices versus fixed CPAP reported in the same study. Base AHI: baseline apnea-hypopnea index (events/hour) in fixed CPAP group; Base AI: baseline arousal index (events/hour) in fixed CPAP group; fixed = fixed CPAP.

**Figure 6:**
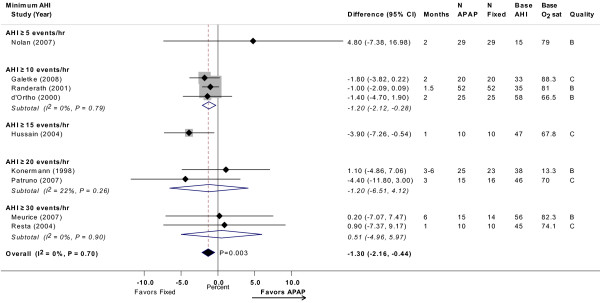
**Minimum oxygen saturation (%) with APAP versus fixed CPAP: meta-analysis, with subgroup analyses by minimum AHI threshold**. See Figure 2 legend. Note that studies favoring APAP are to the left of the vertical 0 line. Senn A and Senn B were comparisons of two different APAP devices versus fixed CPAP reported in the same study. Base AHI: baseline apnea-hypopnea index (events/hour) in fixed CPAP group; Base min O2: baseline minimum oxygen saturation (%) in fixed CPAP group; fixed: fixed CPAP.

Meta-analysis of nine trials showed a non-significant difference in arousal index of -0.85 events/hour (95% CI, -2.2 to 0.5 events/hour; *P *= 0.23), favoring APAP (Figure 5) [10,12,14,20,24,26,29,31,32]. Meta-analysis of nine trials showed a statistically significant difference in minimum oxygen saturation of -1.3% (95% CI, -2.2 to -0.4%; *P *= 0.003), favoring CPAP (Figure 6) [12-14,16,20,24,26,31,32]. Neither meta-analysis had statistically significant heterogeneity. Meta-regression revealed no differences across AHI or study design subgroups.

The three trials reporting on sleep efficiency (percentage of time asleep while in bed) after a period of treatment did not find a statistically significant difference between APAP and CPAP for improvement in the percentage of time spent asleep [14,24,31]. Nine trials reporting on percentage of time spent in REM sleep did not find statistically significant differences between groups [13,14,20,24,26,27,29,31,32]. Seven of eight trials found no statistically significant difference in slow wave sleep (stages 3 or 4) [13,14,20,24,26,27,31,32]. The one outlier reported a statistically significant net mean increase of 7.8% (95% CI, 1.8 to 13.7%; *P *< 0.01); patients using APAP spent more time in slow wave sleep [31]. No study reported effect on the multiple sleep latency test.

### Quality of life

Nine trials evaluated quality of life measures [11,13,15,17,19,22,25,30,33]. One trial that included the SF-36^® ^(Short Form Health Survey) found a significant difference in the mental health (net difference of 5 points; 95% CI, 0.2 to 9.8 points; *P *< 0.05) and vitality (net difference of 7 points; 95% CI, 0.6 to 13.4 points; *P *< 0.05) components, favoring those who used APAP [22]. No other significant differences in quality of life measures between APAP and CPAP were reported in this or the other eight trials. One trial reported on the Sleep Apnea Quality of Life Index and found no difference between groups [33]. No study reported any effect on the Functional Outcomes Sleep Questionnaire. Due to the heterogeneity of specific quality of life outcome reported (13 among these studies, including the components of SF-36), in Table 10 we summarized only whether any measure statistically favored APAP or CPAP and if so, what the net difference was.

### Blood pressure

Three trials reported changes in blood pressure [14,16,33]. Two studies did not find significant differences in blood pressure changes between the APAP and CPAP groups [14,33]. In the third study [16], based on reported data, we estimated a non-significant greater reduction in systolic blood pressure (net difference = 6 mmHg; 95% CI, -1 to 13 mmHg; *P *= 0.09) and a significant greater reduction in diastolic blood pressure (net difference = 8 mmHg; 95% CI, 4 to 11 mmHg; *P *< 0.001) with CPAP compared to APAP.

### Adverse effects

No trials reported any unexpected adverse effects with positive airway treatments. Seven trials reported quantitative comparisons of treatment-related side effects between groups [12,14,15,17,19,25,26]. The side effects were dry mouth, air leakage, skin or nasal-oral irritation. No differences in side effects were reported in five trials [12,14,15,25,26]. Two trials reported that the use of APAP was associated with significantly fewer treatment-related side effects [17,19].

## Discussion

Despite the lack of evidence on objective clinical outcomes, given the largely similar magnitude of effects between APAP and CPAP on sleep measures and wakefulness assessment and the relatively small increase (even though statistically significant) in compliance of about 11 minutes with APAP, we concluded that the overall strength of evidence is moderate that APAP and CPAP result in largely similar treatment effects for patients with OSA (Table 11).

**Table 11:** Strength of evidence of APAP versus CPAP

Outcomes	Number of trials	Total number	**Trials with data for M**eta-analysis	**M**eta-analysis **results comparing APAP with CPAP**	Favors	Strength of evidence
Clinical outcomes (death, cardiovascular events and others)	0	0	0	N/A		Insufficient
Compliance	24	1008	22	0.18 hours (95% CI 0.05 to 0.31; *P *= 0.006)	APAP	Moderate
Apnea-Hypopnea Index	16	548	16	0.25 events/hour (95% CI -0.16 to 0.66; NS)	No difference	Moderate
Epworth Sleepiness Scale	22	954	18	-0.48 (95% CI -0.81 to -0.15; *P *= 0.005)	APAP	Moderate
Arousal Index	10	356	9	-0.85 events/hour (95% CI -2.2 to 0.5; NS)	No difference	Moderate
Minimum O_2 _saturation	9	258	9	-1.3% (95% CI -2.2 to -0.4; *P *= 0.003)	CPAP	Moderate
Sleep efficiency	3	126	0		No difference	Insufficient
Rapid eye movement sleep	9	273	0		No difference	Moderate
Slow wave sleep	8	243	0		No difference in seven trials; one trial favored APAP	Moderate
Quality of life	9	509	0		No difference in eight trials; one trial favored APAP	Moderate
Blood pressure	3	149	0		No difference in two trials; one trial favored CPAP (decrease in diastolic blood pressure)	Insufficient

APAP: auto-titrating CPAP; CPAP: continuous positive airway pressure; N/A: not applicable; NS: non-significant.

The aim of this study was to systematically compare the treatment effects of APAP versus fixed CPAP. Twenty-four trials that included over 1,000 patients provided evidence that APAP reduces sleepiness as measured by ESS by approximately 0.5 points more than fixed CPAP. For compliance, there was a statistically significant difference of 11 minutes per night also favoring APAP compared to fixed CPAP. The clinical significance of these reported improvements in ESS and compliance, however, is unclear. The two types of devices were found to result in similar changes from baseline in AHI, quality of life and most other sleep study measures. However, there is also evidence that minimum oxygen saturation improves more with CPAP than with APAP by about 1%. Evidence is limited regarding the relative effect of fixed CPAP and APAP on blood pressure. There were no data on objective clinical outcomes.

The etiology and severity of OSA varies widely across patients, as do patients' symptoms and their ability to tolerate or consistently use treatments. CPAP, specifically, can be cumbersome and uncomfortable to use; therefore, it is of particular importance to identify which subgroups of patients may benefit most from which specific treatments to maximize the effectiveness of intervention. Our subgroup meta-analyses based on different minimum AHI thresholds to define OSA failed to demonstrate any difference in effectiveness between APAP and CPAP for all outcomes. Higher AHI is used as a marker for more severe disease [35] and is associated with greater mortality [1,36-38]; however, our power to find any differences based on baseline AHI was low, particularly since the study eligibility categories overlapped. Even if we had found a difference, it would at best be hypothesis-generating and would need to be confirmed in a primary study. But we found no study that directly analyzed any subgroup of patients who may particularly benefit from a given treatment. It should be noted that experts have opined that APAP may be used in the setting of failed fixed CPAP (CD, personal communication), although we are not aware of a study in such a setting.

Despite the addition of newer studies in our meta-analyses, our findings differed little from those of a Cochrane meta-analysis reported in 2009, which reviewed 30 randomized trials enrolling 1,136 patients in total (the Cochrane review had a larger number of RCTs because it included results from posters and conference proceedings) [4]. The Cochrane review segregated its analysis by study design into cross-over and parallel design studies but did not provide an aggregate analysis combining both types of study design. In that meta-analysis, a statistically significant difference in compliance of 12.6 minutes per night (95% CI, 4.8 to 21 minutes increase) was found in favor of APAP in cross-over studies, but no significant difference in parallel design studies. It also reported a statistically significant decrease in ESS of 0.64 points (95% CI, 0.12 to 1.16 point decrease) in favor of APAP in cross-over studies, but no significant difference in parallel design studies. We reported similar findings for the two study designs in our full technical review [6].

Follow-up durations in the studies reviewed tended to be short, in the order of weeks to a few months, and are clearly insufficient for the appraisal of the treatment of a life-long disease whose clinical sequelae may take decades to develop. Study dropout rates were also frequently very high, particularly given the short duration of follow-up. In some studies, up to 40% of participants were lost to follow-up within weeks. The ability to meaningfully interpret the findings from these studies is clearly diminished. Other frequent methodological problems included incomplete reporting and/or inadequate analyses. In particular, relatively few studies provided the net differences between interventions (in parallel design studies) or the difference between final values with appropriate adjustments for correlation (in cross-over studies) with their confidence intervals and *P *values. Thus for the large majority of studies, we had to estimate the confidence intervals of the differences between interventions. We also did not search for unpublished and non-English language studies.

## Conclusions

APAP and CPAP were similar in affecting relatively short-term changes in AHI, quality of life, and most other sleep study measures in the treatment of patients with moderate to severe OSA but without significant comorbidities. APAP, however, did reduce sleepiness by approximately 0.5 ESS points more than fixed CPAP. Patients who received APAP also had objectively measured compliance of 11 minutes per night more than those who received fixed CPAP. We surmise that the clinical significance of these reported improvements in ESS and compliance is marginal at best. It is doubtful that additional short-term trials comparing APAP and CPAP to examine these measures will substantially alter these results. However, longer-term and larger trials that evaluate clinical outcomes, such as cardiovascular events, and directly estimate differential effects in different sub-populations may be of value. Furthermore, the current trial evidence is limited to patients newly diagnosed with sleep apnea or who are otherwise naïve to CPAP; thus, future trials of patients who had previously used CPAP may be of value. For now, based on the available data from experimental studies on short-term effects only, the decision to use APAP versus CPAP may well depend on individual patient preferences, specific reasons for non-compliance, costs and other practical considerations that clinicians and patients will need to assess on an individual basis.

## Abbreviations

AHI: apnea-hypopnea index; AHRQ: Agency for Healthcare Research and Quality; APAP: auto-titrating positive airway pressure; CPAP: fixed continuous positive airway pressure; ESS: Epworth Sleepiness Scale; OSA: obstructive sleep apnea; RCT: randomized controlled trial; REM: rapid eye movement; SF-36^®^: Short Form Health Survey.

## Competing interests

The authors declare that they have no competing interests.

## Authors' contributions

SI and EMB designed the study, extracted and analyzed the data and drafted the manuscript. CD provided domain expert input, and reviewed and edited the manuscript. KP, NO, GDK and MC extracted and analyzed the study data, and also reviewed and edited the manuscript. All authors read and approved the final manuscript.
